# Identification, Diversity and Evolution of MITEs in the Genomes of Microsporidian Nosema Parasites

**DOI:** 10.1371/journal.pone.0123170

**Published:** 2015-04-21

**Authors:** Qiang He, Zhenggang Ma, Xiaoqun Dang, Jinshan Xu, Zeyang Zhou

**Affiliations:** 1 College of Life Sciences, Chongqing Normal University, Chongqing, China; 2 State Key Laboratory of Silkworm Genome Biology, Southwest University, Chongqing, China; University of Ottawa, CANADA

## Abstract

Miniature inverted-repeat transposable elements (MITEs) are short, non-autonomous DNA transposons, which are widespread in most eukaryotic genomes. However, genome-wide identification, origin and evolution of MITEs remain largely obscure in microsporidia. In this study, we investigated structural features for *de novo* identification of MITEs in genomes of silkworm microsporidia *Nosema bombycis* and *Nosema antheraeae*, as well as a honeybee microsporidia *Nosema ceranae*. A total of 1490, 149 and 83 MITE-related sequences from 89, 17 and five families, respectively, were found in the genomes of the above-mentioned species. Species-specific MITEs are predominant in each genome of microsporidian Nosema, with the exception of three MITE families that were shared by *N*. *bombycis* and *N*. *antheraeae*. One or multiple rounds of amplification occurred for MITEs in *N*. *bombycis* after divergence between *N*. *bombycis* and the other two species, suggesting that the more abundant families in *N*. *bombycis* could be attributed to the recent amplification of new MITEs. Significantly, some MITEs that inserted into the homologous protein-coding region of *N*. *bombycis* were recruited as introns, indicating that gene expansion occurred during the evolution of microsporidia. *NbS31 *and *NbS24 *had polymorphisms in different geographical strains of *N*. *bombycis*, indicating that they could still be active. In addition, several small RNAs in the MITEs in *N*. *bombycis* are mainly produced from both ends of the MITEs sequence.

## Introduction

Miniature inverted-repeat transposable elements (MITEs) were originally discovered in maize genome, and subsequently found in most eukaryotic genomes, including rice, Arabidopsis, mosquitoes, silkworm and humans [1–5]. MITEs are non-autonomous DNA transposons, and have many common characteristics: terminal inverted repeat (TIR) and target site duplication (TSD); high AT content; high copies. Despite their inability to encode transposase, MITEs can transpose using transposase from other autonomous DNA transposons [6–10]. MITEs play an important role in gene expression and genome evolution [2, 3, 11–14]. Previous study showed that rice *mPing* elements provide new binding sites for transcription factors or other regulatory proteins to significantly increase the expression level of the gene near *mPing* insertion [12]. Abundant copies of MITEs usually affect genomic size, and diversify genotype polymorphism loci in the genome [15, 16]. MITE-derived small RNAs can regulate specific target genes at the transcriptional and post-transcriptional levels [17, 18]. Transposition of MITEs has been demonstrated in many species, including *mPing* and *mGing* in rice, *MADE1* in human cell culture, *mimp1* in fungus and *dTstu1* in potato [9, 19–22]. Therefore, MITEs were considered as sources of genetic material to transfer heterogeneous gene. For instance, rice Stowaway element has been used as a genetic engineering tool to deliver cargo genes into the yeast genome [23].

Microsporidia are obligate intracellular parasitic fungi that can infect a wide range of organisms, including vertebrates and invertebrates (particularly insects). Some species can cause severe diarrhea, encephalitis and hepatitis in Acquired Immunodeficiency Syndrome (AIDS) patients infected with microsporidia [24, 25]. *Nosema bombycis*, the earliest identified species of microsporidia, is a fungal pathogen causing pebrine disease, which can lead to devastating economic losses in the silkworm industry. *N*. *bombycis* contains large amounts of repetitive elements, which comprise about 25% of the whole genome [26]. *Nosema antheraeae* is an obligatory parasite to free-range silkworms *Antheraea pernyi*, and is closely related to *N*. *bombycis* [26, 27]. *Nosema ceranae* is a common pathogen of western honeybee (*Apis mellifera*), and was thought to be relevant to colony collapse disorder [28]. Since the full-length transposons had been firstly reported in microsporidia *N*. *bombycis* [29], a large amount and various types of transposons have been identified continuously in microsporidia genomes based on high-throughput sequencing technique. Up to now, there were at least seventeen microsporidia species genomes have been known to contain transposons, including the most insect-pathogenic species, Nematoda-pathogenic species, mariner-pathogenic species and so on [26, 30–37]. Transposons have become important components facilitated by host-parasite interaction in microsporidia. Several studies have indicated that HGT from metazoan host should be the main origin of several transposons in microsporidian genomics, such as *piggyBac* family *NbPB1*, Helitron family *NbLep1* and *Mariner/Tc1* superfamilies [26, 33, 38]. Although many types of transposons are reported in various microsporidia species mentioned above, studies on MITEs were seldom performed at the whole genome level. The distribution, origin and evolutionary history of MITEs among these related Nosema species remain unknown. A recent report showed that in *N*. *bombycis* genome, MITEs have destroyed the copy of small ribosomal subunits but are still transcribed together with the latter [39], indicative of penitential association with gene structure variation.

In this study, genome-wide identification of MITEs was performed on the whole genomes of microsporidian *N*. *bombycis* and *N*. *antheraeae*, as well as their distantly related species, *N*. *ceranae*. Classification and polymorphism of MITEs and their roles in genomic evolution are discussed, which could contribute to further understanding of MITE function in microsporidia.

## Materials and Methods

### Data and material resources

Genomic sequences and gene annotation information of *N*. *bombycis* CQ1 and *N*. *antheraeae* YY were freely downloaded from public Silkworm Pathogen Database (SilkPathDB, http://silkpathdb.swu.edu.cn/silkpathdb/ftpserver), or can be downloaded from Genbank (Accession no. ACJZ01000001-ACJZ01003558) and S1–S2 File. Information on *N*. *ceranae* genome was obtained from Genbank accession no.ACOL01000001-ACOL01005465. Considering the genes annotation of *N*. *bombycis* and *N*. *antheraeae* might exist a potential over-prediction of small genes, re-annotating the genes in *N*. *antheraeae* and *N*. *bombycis* were fulfilled referring to methods described in previous study [32]. Base on this method that taking advantage of transcriptional signals close to translation initiation sites (TIS) in 5’ upstream of AUG initiation codons, the re-annotating procedure for *N*. *bombycis* and *N*. *antheraeae* was executed as following: CCC-like or GGG-like motifs in the 30 nts upstream and downstream of AUG initiation codons from previous annotation of CDSs were searched to revise the start codons, and then AT content >80% was supplemented as an additional criterion to ensure this revision [32]. Finally, there were 359 over-prediction genes in *N*. *bombycis* and 202 over-prediction genes in *N*. *antheraeae* have been rectified and then used in this study. The information of re-annotation genes for *N*. *bombycis* and *N*. *antheraeae* were both listed in S1 Table.

Four strains of *N*. *bombycis* involved in this study were kindly provided by Dr Pan Guoqing (Southwest University, Chongqing Province, China), Boling Yue, Sericulture Research Institute, Guangxi Province, China), Dr. Qiong yang (Sericulture & Agri-food Research institute GAAS, Guangdong Province, China), Shiliang Chen (Sericultural and Apicultural Institute, Yunnan Province, China), which were labeled as strains CQ1, GX, GD and YN, respectively. All four strains were isolated from the local silkworm and reserved in their own labs mentioned above.

### Identification of MITEs from microsporidian Nosema

Based on the structure of MITEs, MITE-Hunter was used to identify MITE candidates from *N*. *bombycis*, *N*. *antheraeae and N*. *ceranae* genomes [40]. MITE-Hunter parameters were set as follows: TIR: 5-60bp; TSD: 2-20bp; length <1500bp; TIR mismatch <2. After filtering sequences of candidate MITEs that contain <3 N and TSD mismatches, those elements that shared ≧90% sequence similarity by blast-all were considered to belong to the same MITE family. The families containing less than three members were discarded from this study.

To mine all the remnant copies of each MITE family, the conserved sequences from each family were used as the query sequence to scan each genome sequence by homology BLAST search. The false results were filtered out based on the criteria of nucleotide identity rate <90% and query coverage <80%. All MITE families were then searched against the RepBase (version 18.05) [41] and Genbank database, respectively, to be classified as known and unknown families. The MITE families were also assigned into superfamilies based on their similarities of TIR and TSD sequences. Each MITE family identified here was designated as NxY#, where x is a letter representing the microsporidian species, Y is a letter representing a superfamily and # is a number representing the family. Letter S stands for *Stowaway-like*, T for *Tourist-like*, h for *hAT-like*, Me for *Merlin-like*, Mu for *Mutator-like*, and N for others.

### Sequence analysis and phylogenetic construction

The copy elements of each MITE family were aligned using MUSCLE [42], and neighbor-joining trees were constructed using MEGA5 [43]. Likewise, the nucleotide divergence for each MITE family was calculated using MEGA with the Kimura2-parameter model. The expected hairpin structures for representative intact MITEs were predicted using the mfold web server (http://mfold.rna.albany.edu/). To find small RNAs derived from the MITE transposons, the elements from each MITE family were used as the query sequence to scan the *N*. *bombycis* small RNA database by Bowtie2.0, with mismatch <2 [44]. These MITE-derived small RNAs were mapped to the canonical MITE sequences to evaluate their derived position in MITEs.

### Analysis of presence/absence of MITE polymorphisms

The presence/absence of a MITE in a particular region can produce polymorphism between the relatives, so the different isolates from the same species can be distinguished by MITE insertion polymorphism (MIP) analysis [7]. Accordingly, the presence/absence of polymorphism due to specific MITEs in four different geographical strains of *N*. *bombycis* was explored by MIP analysis. Firstly, orthologous genes between *N*. *bombycis* and *N*. *antheraeae* were acquired by reciprocal best-hits BLAST. Then, these orthologous genes in a collinear gene order were considered as syntenic regions in respective species. Subsequently, these shared syntenic regions were used to investigate whether there were the shared syntenic regions harboring MITE elements in the *N*. *bombycis*, but harboring no MITE elements in the *N*. *antheraeae*. Ultimately, eighteen shared syntenic regions where MITEs had inserted in the 5’-flanking regions of orthologous genes from *N*. *bombycis* but not inserted in the same region from *N*. *antheraeae* were selected as candidates to execute the subsequent PCR analysis (S1 Fig). The primer pairs used for PCR amplification were designed according to these candidate regions harboring MITEs and homologous genes. Primer information and expected product size, generated by Primer 5.0, are listed in S2 Table. Consequently, the molecular size of PCR products for several different geographical isolates of *N*. *bombycis* can be judged for the presence/absence of polymorphism due to specific MITEs in those syntenic regions mentioned above. The genomic DNA of the spores was extracted using the CTAB (cetyltrimethylammonium bromide) method. PCR was performed as follows: 5 min at 94 ^o^C, 35 cycles of 95 ^o^C for 40 sec, 53–59 ^o^C (dependent on the primers) for 40 sec, and 72 ^o^C for 1 min, with a final 5-min extension at 72 ^o^C. The PCR products were separated on 1% agarose gels, stained with ethidium bromide and visualized on a UV trans-illuminator.

### Small RNA library preparation and sequencing

Silk glands and whole bodies of day-3 fifth instars larvae infected with *N*. *bombycis* were collected for RNA isolation. Following purification, total RNA samples were instantly preserved in ethanol and stored at -80 ^o^C until further use. For deep sequencing, the small RNA samples were prepared as follows: total RNA of each sample was size fractionated on a 15% PAGE gel, and small RNAs around 16–30 nt in length was collected. The 5’RNA adapter (5’-GUUCAGAGUUCUACAGUCCGACGAUC-3’) was ligated to the RNA pool with T4 RNA ligase. Ligated RNA was size fractionated on a 15% agarose gel, and a 40–60 nt fraction excised. The 3’RNA adapter (5’-pUCGUAUGCCGUCUUCUGCUUGidT-3’; p, phosphate; idT, inverted deoxythymidine) was subsequently ligated to precipitated RNA using T4 RNA ligase. Ligated RNA was size-fractionated on a 10% agarose gel, and the 70–90 nt fraction (small RNA + adaptors) excised. Small RNAs ligated with adaptors were subjected to RT-PCR (Superscript II reverse transcriptase, 15 cycles of amplification) to produce sequencing libraries. PCR products were purified and small RNA libraries were sequenced using Solexa, a massively parallel sequencing technology.

## Results

### Identification of MITEs in three Nosema genomes

Based on the common structure of MITEs, genome-wide search using the MITE-Hunter program yielded 409, 69 and 31 candidate MITE families from *N*. *bombycis*, *N*. *antheraeae* and *N*. *ceranae*, respectively. After filtering out false-positive results (see Materials and Methods), 89, 17 and five families of MITEs were finally included. The length of canonical elements (defined as full-length elements with a TSD and ITR) varied among different families, with TSD length range from 2 to 10 bp and TIR length range from 5 to 34 bp (Table 1 and S3 Table). Based on TIR and TSD sequences, some families identified in this study were classified as known superfamilies including *Stowaway-like*, *Tourist-like*, *hAT-like*, *Merlin-like* and *Mutator-like*, while others were classified as new unknown superfamilies. The characteristics of each superfamily are shown in Table 1. Notably, among the new families in *N*. *bombyci*s, *N*. *antheraeae* and *N*. *ceranae*, there was a common family whose TSD was 5-6bp in length and TIR sequence started with TGT (S2 Fig), which is similar to *MiS5* and *MiS22* of *Solanaceae* [17].

**Table 1:** Summary of MITE Superfamilies in Microsporidia.

Superfamily	Family Number			Total Elements			Length of All Elements (bp)			TSD	TIR (bp)
	Nb	Na	Nc	Nb	Na	Nc	Nb	Na	Nc		
*Stowaway-like*	32	3	1	482	21	12	2.19×10^5^	2.76×10^3^	1.55×10^3^	TA	5–30
*Tourist-like*	19	7	3	229	65	46	1.08×10^5^	1.05×10^4^	6.88×10^3^	3bp	5–26
*hAT-like*	8	1	0	309	8	0	9.22×10^4^	2.15×10^3^	0	8bp	5–18
*Merlin-like*	4	0	0	48	0	0	2.1×10^4^	0	0	8bp	24–34
*Mutator-like*	1	0	0	9	0	0	1.08×10^3^	0	0	10bp	5
Others	25	5	1	413	55	25	1.24×10^5^	1.00×10^4^	7.67×10^3^	4-7bp	5–28
Tatal	89	17	5	1490	149	83	5.66×10^5^	2.54×10^4^	1.11×10^4^		

NOTE: Nb: *Nosema bombycis*, Na: *Nosema antheraeae*, Nc: *Nosema ceranae*.

After scanning the three Nosema genome sequences, copy numbers of each MITE family were estimated. The genomes of *N*. *bombycis*, *N*. *antheraeae* and *N*. *ceranae* harbor 1490, 149 and 83 MITE-related sequences, constituting 3.5%, 0.51% and 0.15% of the total genome, respectively (Table 1).The information of all families’ sequences of MITEs and its distribution in each of three Nosema genomes can be seen in S4 Table and S3–S5 File. AT content was 47–79% in the sequence of each MITE family, which is common for MITEs. *Nbh4* was the most abundant in *N*. *bombycis*, with 122 copies in the genome including 112 intact copies (S3 Table). *NbT2*, *NbT8* and *NbS2* in *N*. *bombycis* share high sequence homology with *NaT8*, *NaT6* and *NaS2* in *N*. *antheraeae*, respectively (S3 Fig), indicating that *NbT2* and *NaT8*, or *NbT8* and *NaT6*, or *NbS2* and *NaS2* must have originated from a common ancestor before the divergence between *N*. *bombycis* and *N*. *antheraeae*. However, no MITE family in *N*. *ceranae* shared homology with any MITE family from *N*. *bombycis* or *N*. *antheraeae*.

### Some MITE families in *N*. *bombycis* have undergone multiple rounds of rapid amplifications

**Fig 1:**
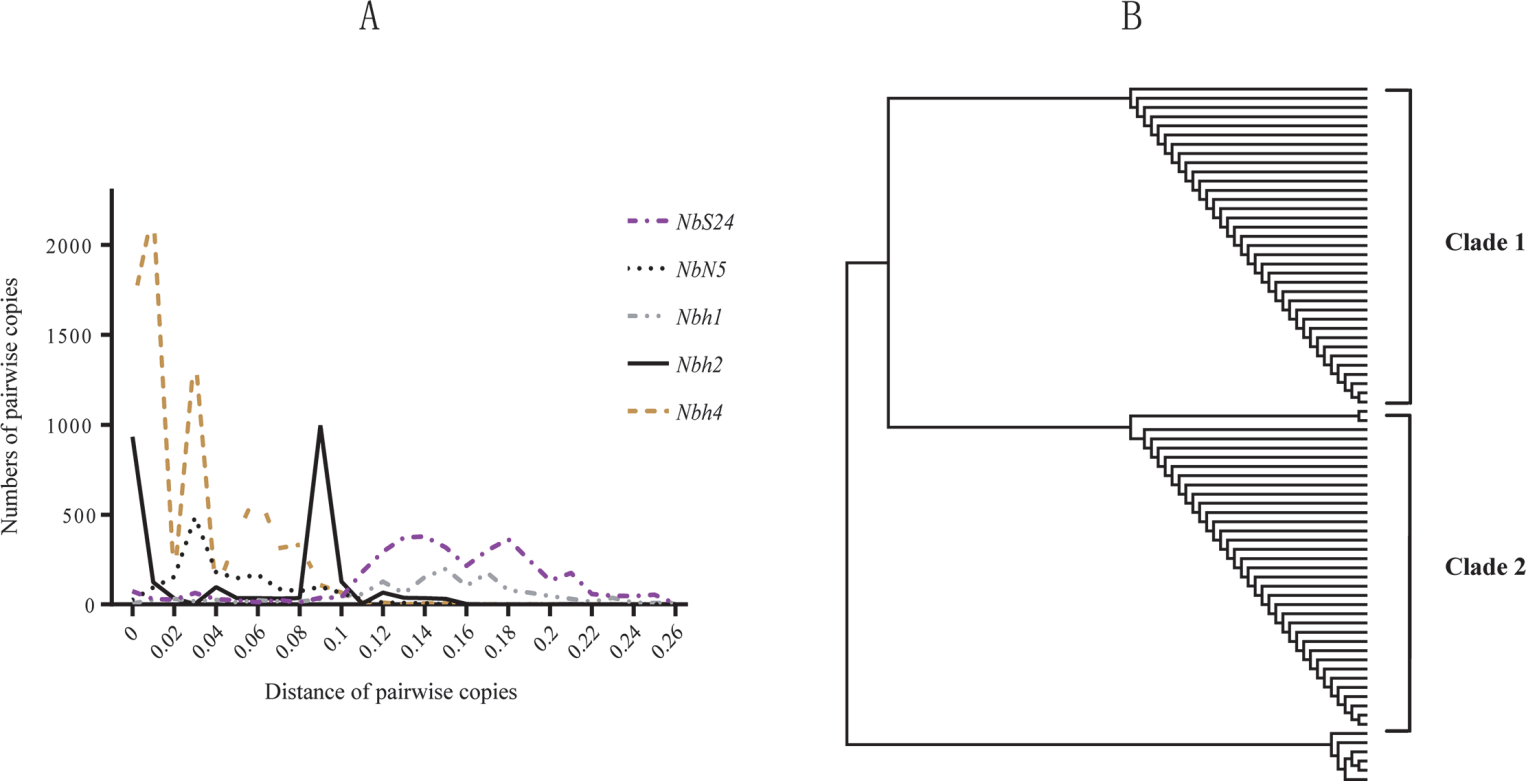
Several rounds of amplifications of MITE families *in N*. *bombycis*. (A) The distribution profile of genetic distance for copies of MITE families. X-axis represents the interval of genetic distance; Y-axis represents the numbers of any pairwise copies. (B) The tree diagram for the copies of *Nbh2* families with neighbor-joining (NJ) method.

To further investigate the mechanisms of MITEs amplifications in *N*. *bombycis*, sequence diversity was analyzed for those elements containing more than 50 copies, including *Nbh1*, *Nbh2*, *Nbh4*, *NbN5* and *NbS24*.The histograms for these five MITE families have peaks at different levels of genetic distance, ranging from 0 to 0.26 (Fig 1A), suggesting that the amplification bursts occurred at distinct time points. Notably, for *Nbh2*, dominant amount of the copies distributed in two areas of genetic distance (two peaks, Fig 1A) and phylogenetic trees constructed for *Nbh2* copies were also grouped into two well-supported clades (Fig 1B), indicating that *Nbh2* must have experienced two rounds of rapid amplifications in evolutionary history. *Nbh4* has 122 copies in the genome, including 112 intact copies and 10 fragment copies. The distributions of genetic distance for *Nbh4* members show that they have at least three peaks (Fig 1A). Notably, the 112 intact copies of *Nbh4* can be divided into 24 groups based on 100% nucleic acid sequence identity (S4 Fig). For instance, the largest group consisted of 38 identical elements, while the second group was made up of 29 identical elements. These results suggested that *Nbh4* must have recently experienced multiple rounds amplification.

### Distribution of MITEs in genomes of three Nosema species

The distribution of MITEs in genomes was surveyed to examine whether there is any bias for MITEs to associate with genes. If a MITE inserts into within the 300bp upstream and downstream flanking regions of coding sequences, this MITE was regarded as associate with gene regions. We found that 189 (12.7%), 61 (40.9%), 24 (28.9%) of predicted MITEs inserted into gene regions in *N*. *bombycis*, *N*. *antheraeae* and *N*. *ceranae*, respectively. To determine whether insertions of MITEs associated with gene regions were incidental, a computer simulation was performed [45]. However, after a computer simulation was performed as a negative control, chi-square tests between samples and control suggested that the variations were not statistically significant (S5 Table), implying that MITEs of Nosema species do not preferentially insert into gene regions. When a MITE insertion into within respective 200, 100bp, 0bp upstream and downstream flanking regions of a CDS was assumed to be associate with genes, the patterns of MITE insertion in the genome were also similar to that a MITE within 300bp flanking regions of a CDS (S5 Table). Therefore, all these results suggested that there was no insertion bias of MITE toward gene regions.

**Fig 2:**
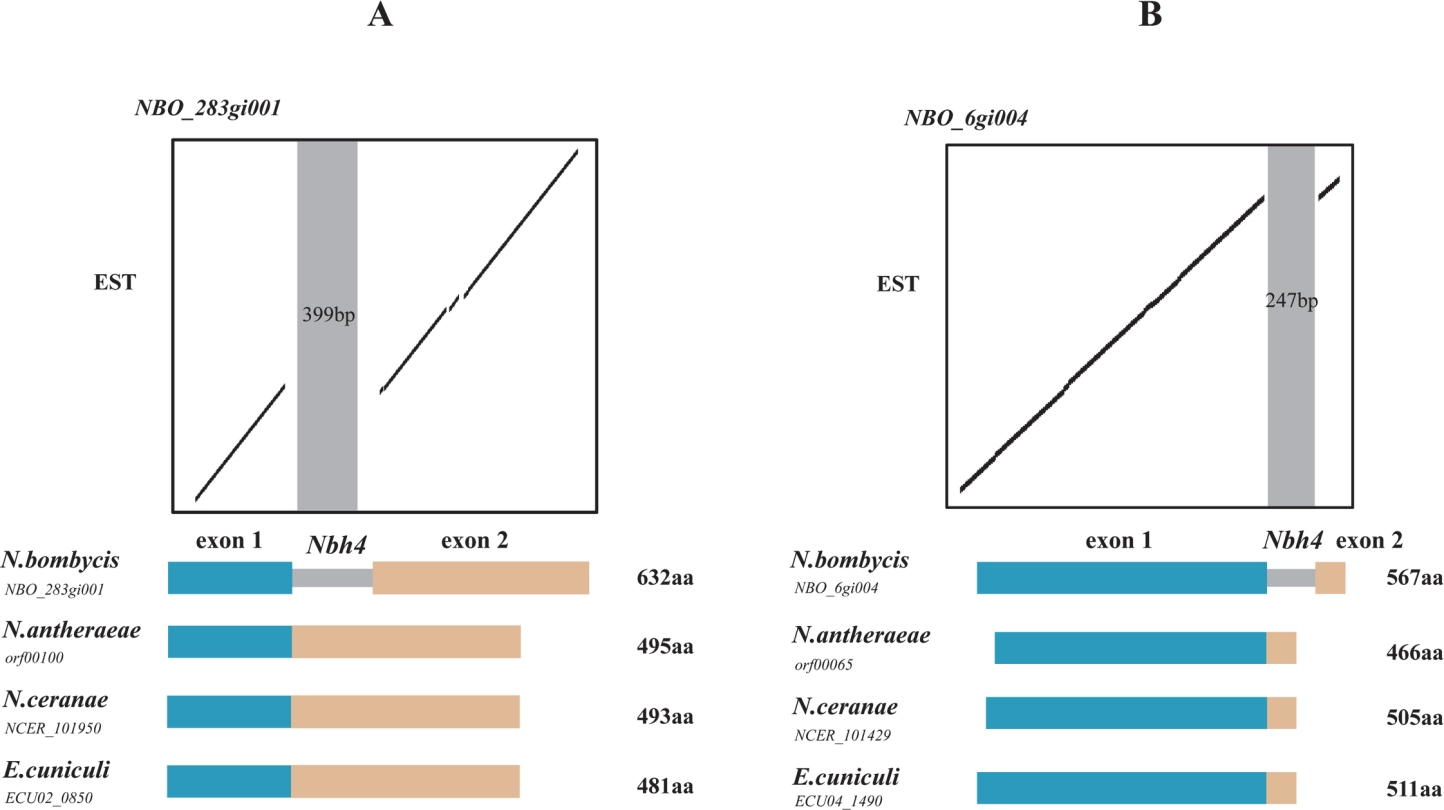
MITEs inserted into protein-coding genes were recruited as introns in *N*. *bombycis*. (A) A dot-plot sequencing comparison of MITE-inserted gene *NBO_283gi001* with its expressed sequences tag. (B) A dot-plot sequencing comparison of MITE-inserted gene *NBO_6gi004* with its expressed sequences tag. The structural comparison of targeted gene and its homologous genes from *N*. *antheraeae*, *N*. *ceranae* and *Encephalitozoon cuniculi* are presented as dot plots, respectively.

To explore the effects of MITEs insertions on structures of protein-coding sequences in *N*. *bombycis*, 21 protein-coding sequences associated with MITEs insertions were targeted to compare the impact of MITEs on gene structure in *N*. *bombycis* with their corresponding homologous genes in *N*. *antheraeae* (Fig 2 and S6 Table). Among these 21 genes, 13 were single-copy genes and eight were one copy of duplicated genes. By BLAT searching of EST database for *N*. *bombycis*, seven single copy genes inserted with MITEs were transcribed except for MITE fragments (Fig 2), indicating that these MITEs were recruited as introns.

### Presence/absence of MITEs polymorphisms in different *N*. *bombycis* geographical strains

**Fig 3:**
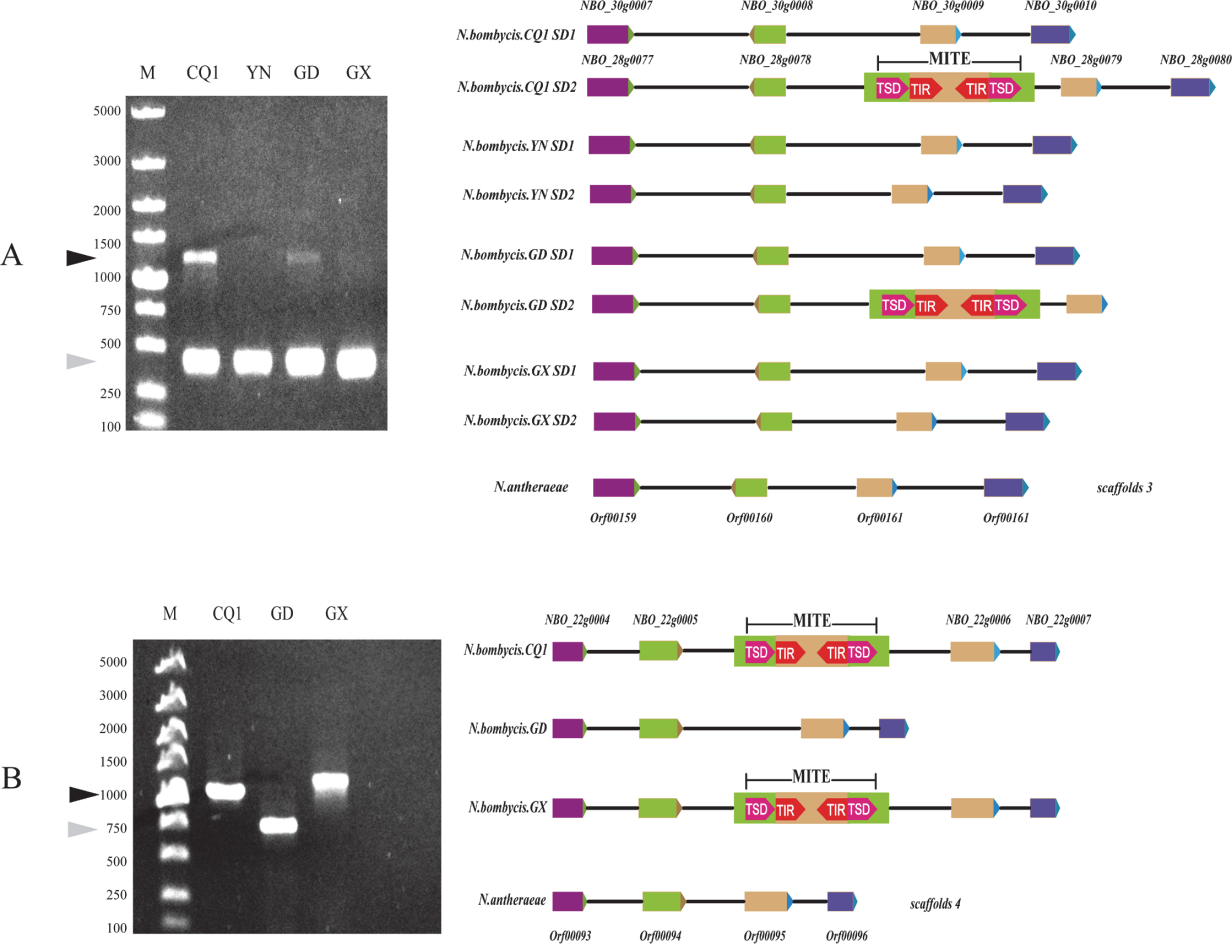
Presence/absence of MITEs polymorphisms in four geographical strains of *N*. *bombycis*. (A) The product of *NbS3*-inserted PCR amplification in four strains, SD1 and SD2 represent one pair of segmental duplication in *N*. *bombycis*. (B) The product of *NbS24*-inserted PCR amplification in three strains and the illustration. M, DNA marker, CQ1: Chongqing isolate, YN: Yunnan isolate, GD: Guangdong isolate, GX: Guangxi isolate. Black arrowhead: MITE-inserted PCR-amplified products. Gray arrowheads: PCR-amplified product without MITEs insertion. Rectangular arrowheads: genes in the syntenic region among several *N*. *bombycis* isolates and *N*. *antheraeae*. Same color rectangles correspond to homologous genes.

Eighteen genomic fragments inserted with certain MITEs in the genome of CQ1 strain were PCR-amplified to detect the presence/absence of MITE polymorphisms by using three other *N*. *bombycis* geographical strains as DNA templates. Since segmental duplication had occurred in the genome of *N*. *bombycis* CQ1 strain [26], two distinct PCR-amplified bands whose size difference is similar to the length of *NbS31* were visualized (Fig 3A), which is in accordance with previous genomic sequence analysis. As a result, the PCR-amplified DNA fragment containing no *NbS31* elements was common in all four strains, while the other DNA fragment harboring the *NbS31* element was only present in CQ1 and GX strains (Fig 3A). This result showed the existence of an interstrain presence/absence of polymorphism of *NbS31* in *N*. *bombycis*. Likewise, the targeted genomic region containing *NbS24* in CQ1 isolate was PCR-amplified for CQ1, GX and GD strains. PCR-amplified DNA fragment containing *NbS24* elements was only present in CQ1 and GX, while DNA fragment containing no *NbS24* elements was present in GD (Fig 3B). Moreover, the size difference in these two PCR-amplified fragments was similar to the length of *NbS24*. Therefore, *NbS24* of *N*. *bombycis* also has interstrain presence/absence of polymorphism.

### MITE-derived small RNAs

**Fig 4:**
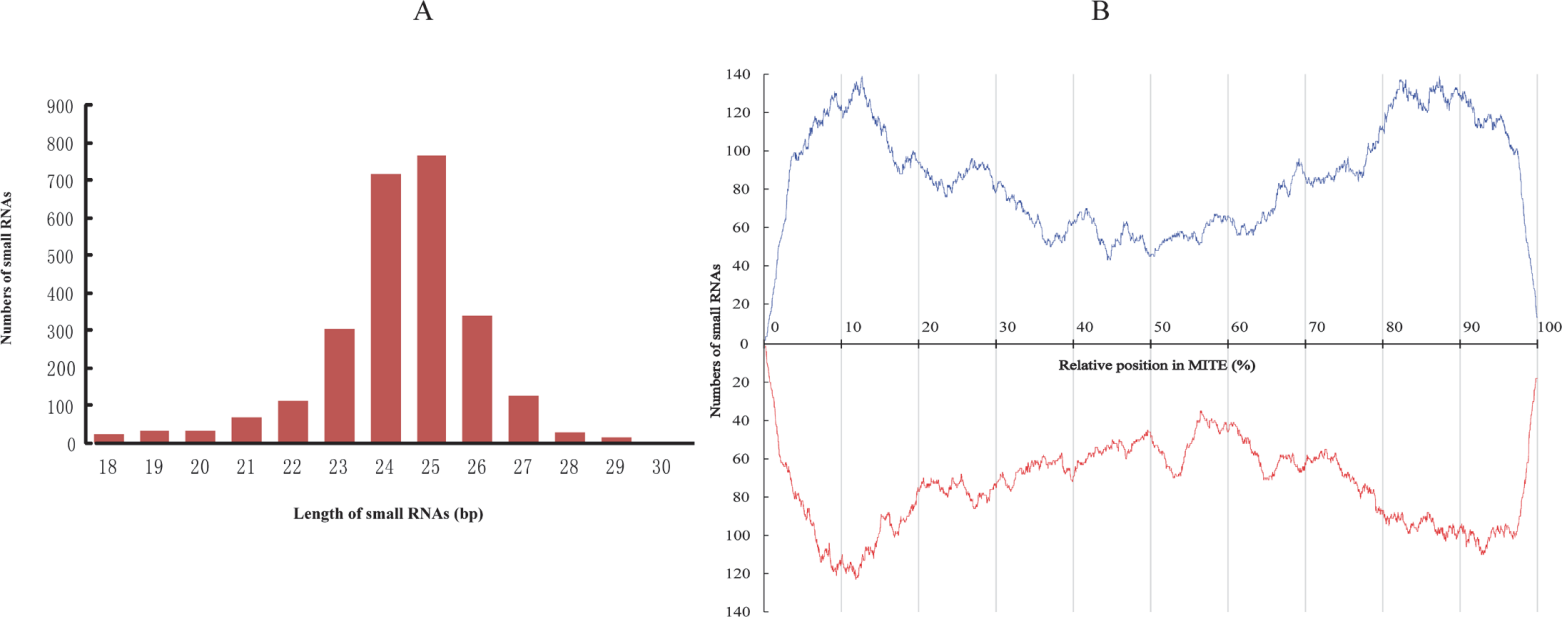
MITE-derived small RNAs in *Nosema bombycis*. (A) Length distribution of small RNAs generated by MITE sequences. (B) Density (sense, black; antisense, red) of small RNA tags assigned to MITE sequences. Frequency is shown along the Y-axis. Relative nucleotide position within the consensus sequence is indicated along the X-axis.

We obtained raw data by sequencing small RNA pools of Silk glands and whole bodies of day-3 fifth instar larve infected with *N*. *bombycis* and filtered the low quality reads, using the sequencing Solexa technology. Finally, a total number of 3,844,786 raw sequence reads were obtained. After filtration, 1,418,249 (36.89%) high-quality reads were remained and 6661,609 reads were matched to *N*. *bombycis* genomic sequence by Bowtie 2.0 software [44]. MITE-derived small RNAs were identified in *N*. *bombycis* by matching all the small RNA tags to the canonical MITEs with Bowtie alignment. About 2,589 non-redundant reads were matched by each MITE family conservative sequence. These MITE-derived small RNAs range from 18–29 nt in length, among which 24–25 nt small RNAs were dominant (Fig 4A). The regions of MITE sequences that can generate small RNAs were also investigated. The result showed that small RNAs with high sequence coverage extended throughout the MITEs, and were mainly derived from two terminals of MITEs (Fig 4B).

## Discussion

In this study, we performed a systematic and genome-wide analysis to search for MITEs in *N*. *bombycis*, *N*. *antheraeae* and *N*. *ceranae*, using an efficient program, MITE-hunter [40]. MITE-hunter, which can identify repetitive sequences with TIR and TSD structures, is an excellent, successful tool for *de novo* identification of MITEs with low false-positive rates as compared to other tools. After removing pseudo-MITEs by a strict filtering process, we identified 89, 17 and five MITE families, respectively. A previous report has shown that *N*. *bombycis* experienced genomic expansion, which was mainly attributed to segmental duplication and a large number of transposons [26]. There were many more MITE families in *N*. *bombycis* as compared to *N*. *antheraeae* or *N*. *ceranae*, which indicated that proliferation of MITEs contributed to genomic expansion in *N*. *bombycis*.

While only three canonical families in *N*. *antheraeae* genome, *NaT8*, *NaT6* and *NaS2*, were found to share homology with those in *N*. *bombycis* genome, remnants of eight MITE families of *N*. *bombycis* also exist in *N*. *antheraeae*. These residual elements indicated that many old MITE families of *N*. *antheraeae* might have lost their activity and cannot amplify, along with accumulation of mutations and deficiency. In contrast, some MITEs in the *N*. *bombycis* genome recently experienced multiple rounds of amplification, and others were newly inserted after divergence of *N*. *bombycis*. Hence, recent insertion and amplification of MITEs must be responsible for the much larger number of MITE families in *N*. *bombycis*. Several MITEs that had recently inserted into the protein-coding genes of *N*. *bombycis* were recruited as introns, suggesting that introns were more generally present in different genes of microsporidia parasite, rather than in ribosomal genes, as previously reported [36]. In addition, although there was no conclusive evidence to show if certain MITEs were still active in *N*. *bombycis*, two MITEs had presence/absence of polymorphisms in several *N*. *bombycis* geographical strains, and their activity could be further explored.

The link between MITEs distribution and spore traits of the three Nosema species still remained unclear. Recent study reported that in domestic silkworm *Bombyx mori*, a partial transposon named as *Taguchi* had inserted in the cis-regulatory region of ecdysone oxidase (*EO*) gene and therein improve its transcribed level, which lead to the more stable developmental phenotype than that silkworm without the transposon insertion when suffering from food shortage [46, 47]. So, the potential function of the Nosema genes that have been targeted by MITE insertion are worth in studying further.

Although it was unclear which factors had driven the amplification of MITEs, "genome shock" theory was proposed to explain the amplification of transposons: vast majority of transposons are not active in the evolutionary history, only external stimuli or catalysis through transposase encoded by homologous autonomous transposon will lead to amplification of MITEs [9, 48]. Studies using yeast expression system have shown that non-MITE ancestral DNA transposons can also catalyze transposition of MITEs [8]. These studies prompted us to analyze the amplification of MITEs in *N*. *bombycis*, since there are many families in this species. Surprisingly, an autonomous *hAT-like* element encoding an intact transposase was found in *N*. *bombycis*, and its TIR sequence is highly homologous to *Nbh4* element, which is the most abundant element in *N*. *bombycis*. So, it can be hypothesized that the recent amplification of *Nbh4* in *N*. *bombycis* could have been catalyzed by a *hAT-like* transposase.

Many studies have shown that organisms have a complete mechanism to reduce transposon activity, such as DNA methylation [49]. Recent studies on plant transposons have shown that a large number of small RNAs originated from MITEs, and could potentially regulate the activity of MITEs [17, 50]. In this study, the MITE-derived small RNAs in *N*. *bombycis* genome were predominantly 24 and 25nt long. Moreover, these RNAs were mainly generated in the position of two-terminal sequence, which was similar to the formation of miRNAs. It would be interesting to examine if these MITE-derived small RNAs in *N*. *bombycis* were generated by a pathway similar to miRNA or siRNA biogenesis. Likewise, the effect of MITE-derived small RNAs on transposons or genes of *N*. *bombycis* deserves further study.

## Supporting Information

S1 Fig The selected genomic fragments used for evaluate presence/absence of MITEs polymorphisms in *N*. *bombycis*. The positions of designed primers were marked as pair of arrows.
(TIF)

S2 Fig Multiple sequence alignment of MITE families whose TSD length is 5-6bp and TIR begin with TGT in *N*. *bombyci*s, *N*. *antheraeae* and *N*. *ceranae*. MITE families of *MiS5* and *MiS22* have been identified in *Solanaceae*.
(TIF)

S3 Fig (A)Structure and homologous regions of three MITEs identified in *N*. *bombycis* and *N*. *antheraeae*. Grey rectangle is TSD, black triangles are TIRs, and white rectangles are homologous regions of each transposon in both species. The corresponding names and percentages of identity are shown on the left.
(TIF)

S4 Fig Sequence alignment of representative *Nbh4* MITE sequences. The copy numbers of identical elements are shown on the left of each sequence. Red arrowheads are TIR.
(TIF)

S1 File The genome sequences of *N*. *antheraeae*. (XLS)

S2 File The gene annotation information of *N*. *antheraeae*. (XLS)

S3 File All the MITE copies sequences identified in *N*. *bombycis* genome. (XLS)

S4 File All the MITE copies sequences identified in *N*. *antheraeae* genome. (XLS)

S5 File All the MITE copies sequences identified in *N*. *ceranae* genome. (XLS)

S1 Table List of the re-annotation genes information in *N*. *bombycis* and *N*. *antheraeae*. (XLS)

S2 Table Primers designed for analyzing presence/absence polymorphisms of MITEs based on *N*. *bombycis* CQ1 genomic sequences. (DOC)

S3 Table Characteristics of all MITE families in three genomes of *N*. *bombycis*, *N*. *antheraeae* and *N*. *ceranae*. (DOC)

S4 Table Distribution information of MITEs in each of three genomes of microsporidian Nosema. (XLS)

S5 Table Chi-square test of biased insertion of MITE associated with gene regions in *N*. *bombycis*, *N*. *antheraeae* and *N*. *ceranae* genomes. (DOC)

S6 Table The information of protein coding regions with MITE insertion in *N*. *bombycis*. (DOC)
