# Supplementary figures and images for: Identification, Diversity and Evolution of MITEs in the Genomes of Microsporidian Nosema Parasites
Source: PLoS One. 2015 Apr 21;10(4):e0123170. doi: 10.1371/journal.pone.0123170 (PMC4405373; doi:10.1371/journal.pone.0123170)

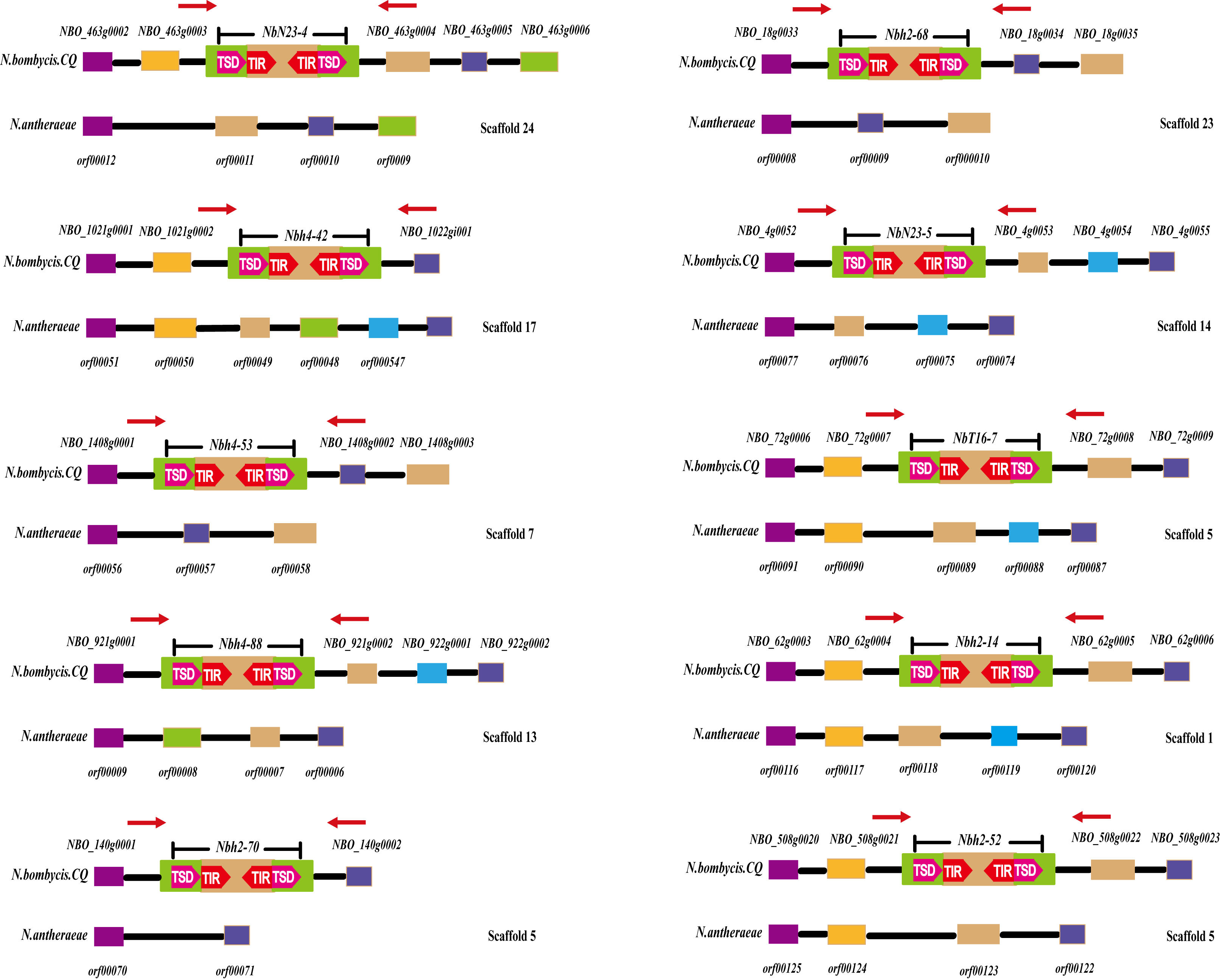

Supplement: S1 Fig — The positions of designed primers were marked as pair of arrows. (TIF) [file pone.0123170.s001.tif]

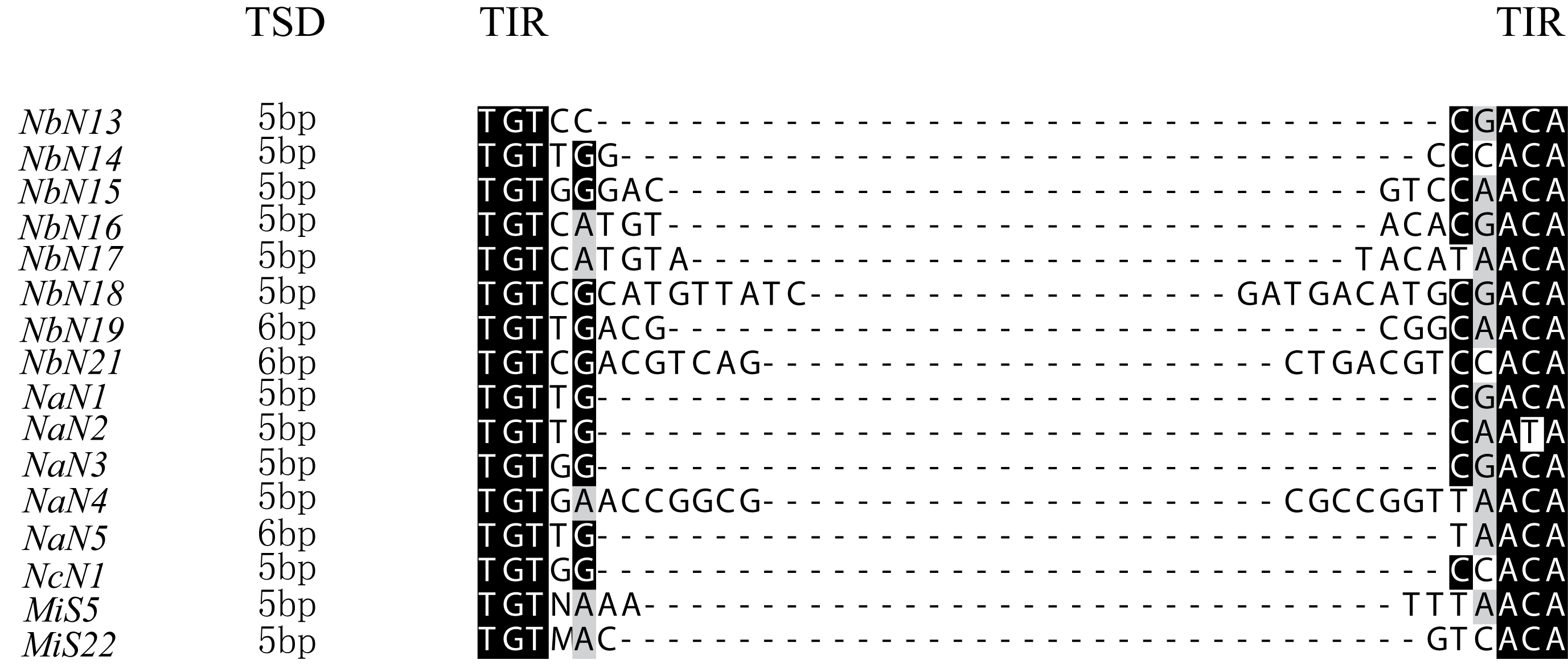

Supplement: S2 Fig — MITE families of MiS5 and MiS22 have been identified in Solanaceae. (TIF) [file pone.0123170.s002.tif]

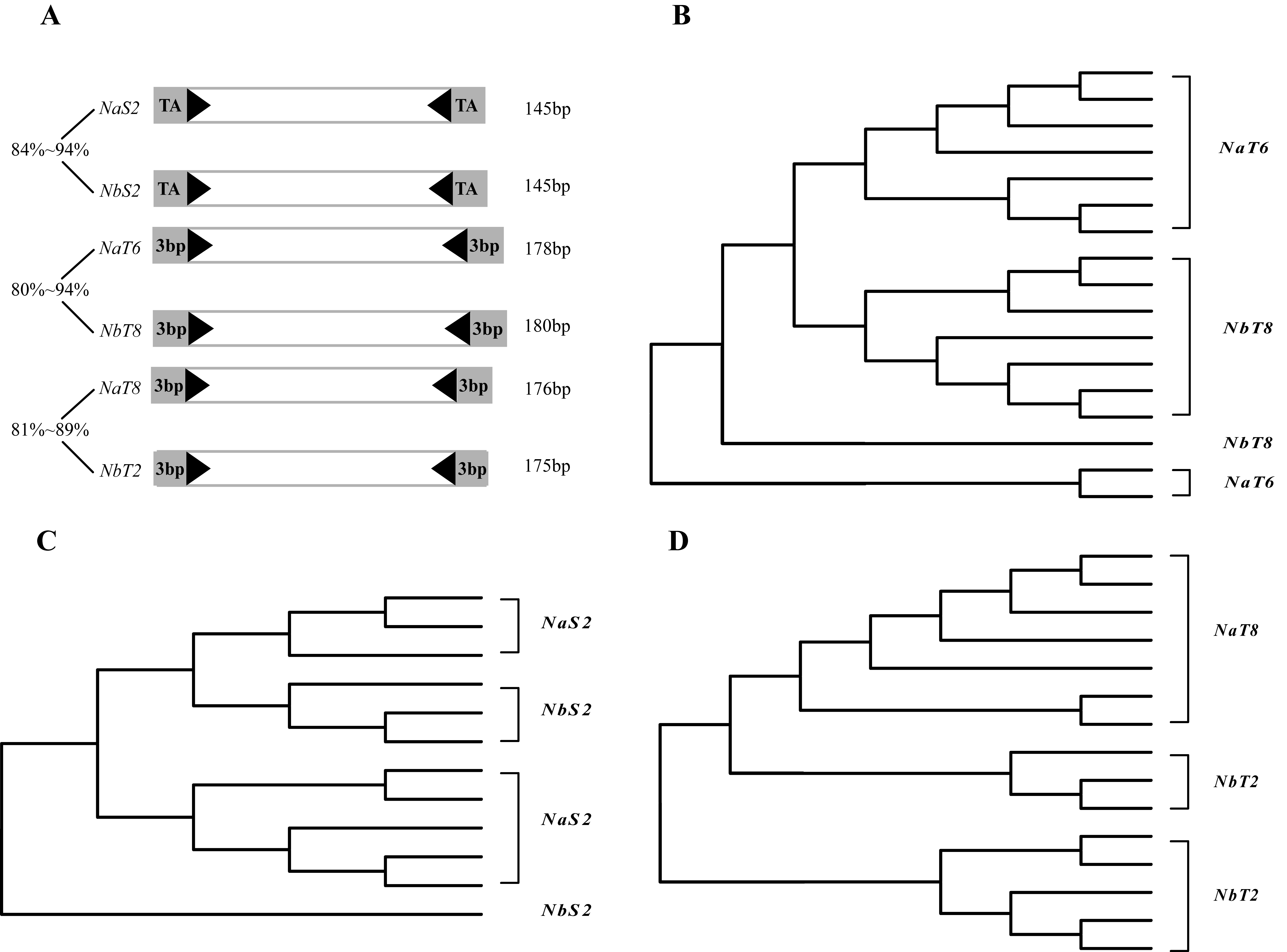

Supplement: S3 Fig — Grey rectangle is TSD, black triangles are TIRs, and white rectangles are homologous regions of each transposon in both species. The corresponding names and percentages of identity are shown on the left. (TIF) [file pone.0123170.s003.tif]

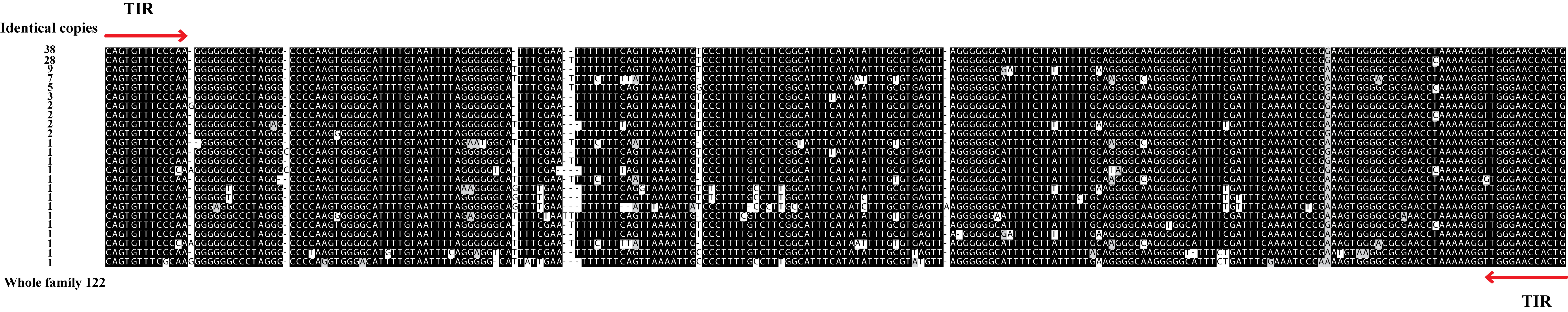

Supplement: S4 Fig — The copy numbers of identical elements are shown on the left of each sequence. Red arrowheads are TIR. (TIF) [file pone.0123170.s004.tif]
