# Supplementary material for: Identification, Diversity and Evolution of MITEs in the Genomes of Microsporidian Nosema Parasites
Source: PLoS One. 2015 Apr 21;10(4):e0123170. doi: 10.1371/journal.pone.0123170 (PMC4405373; doi:10.1371/journal.pone.0123170)
Supplement: S2 Table — (DOC) [file pone.0123170.s011.doc]

| Name | Primers | Sized | Product size | Length of MITEs |
| --- | --- | --- | --- | --- |
| Nbh4-F | 5' TTAAACATACTGCACAACCTCCG 3' | 23bp | 731bp | 242bp |
| Nbh4-R | 5' TTTTGGCTTTCCATAACCGA 3' | 20bp |
| NbS24-F | 5' CAGGGGTGGGCAAACTTTTT 3' | 20bp | 1118bp | 384bp |
| NbS24-R | 5' CTTGGCACAAACTCACCACA 3' | 20bp |
| NbS31-F | 5' TCTTGTGTCATTTGTGGGGTT 3' | 21bp | 1149bp | 795bp |
| NbS31-R | 5' GAAGTTTTCCTTGTGTTTATCCG 3' | 23bp |
| Nbh2-F | 5' TTTAGCCCATGCCCCACTTT 3' | 20bp | 733bp | 214bp |
| Nbh2-R | 5' TCGGTCCTGCATCAAAACTGT 3' | 21bp |
| Nbh4-F | 5' TCAACCACGAAGATGCCAAGA 3' | 21bp | 1133bp | 242bp |
| Nbh4-R | 5' CAAAGATGTAGGAGGGAACCGT 3' | 22bp |
| Nbh2-F | 5' TTTTAGCCCATGCCCCACTT 3' | 20bp | 529bp | 214bp |
| Nbh2-R | 5' ACGGTCATGTCTCCGGTAGT 3' | 20bp |
| NbS24-F | 5' CAAACTTTTTGGGTCGCGGG 3' | 20bp | 1276bp | 349bp |
| NbS24-R | 5' TCCGATCCATGCCATAAAACAG 3' | 22bp |
| NbS31-F | 5' TTTCCTTGTGTTTATCCG 3' | 18bp | 1169bp | 795bp |
| NbS31-R | 5' TCTTCTTGTGTCATTTGTG 3' | 19bp |
| NbT10-F | 5' ATCCGCAGAGTTTGATGGCA 3' | 20bp | 692bp | 212bp |
| NbT10-R | 5' CGCCTTTTTGGGTTTCAGCA 3' | 20bp |
| NbN23-F | 5' AGTGATCCCCCAGGAGTAGG 3' | 20bp | 956bp | 321bp |
| NbN23-R | 5' ACCCTGACTTAACCGCGAAT 3' | 20bp |
| NbS24-F | 5' TTCTTGGCATTCACTGGACCTT 3' | 22bp | 1096bp | 382bp |
| NbS24-R | 5' ACGAGACTGGTTGGTTTTGGT 3' | 21bp |
| NbN23-F | 5' CATGGGTCGGTATGATGGGA 3' | 20bp | 786bp | 321bp |
| NbN23-R | 5' TAGGTAAACGCTCCTTCGGC 3' | 20bp |
| Nbh2-F | 5' AAAAAGTTGGATTTCGGGTGTGT 3' | 23bp | 889bp | 214bp |
| Nbh2-R | 5' TCTCTGCTCTTTTTGGCTGCT 3' | 21bp |
| NbT16-F | 5' TAGTACACCAAGACCCCGCT 3' | 20bp | 1410bp | 924bp |
| NbT16-R | 5' CCACCATCCAACAAATCCCA 3' | 20bp |
| NbN7-F | 5' ATTTCGCTGACCGACTGTGT 3' | 20bp | 1223bp | 387bp |
| NbN7-R | 5' TGACCCAAACTTTTCCTACCGA 3' | 22bp |
| Nbh2-F | 5' TGAAGTTTTCGTGGAGATGATGA 3' | 23bp | 1070bp | 214bp |
| Nbh2-R | 5' AAACGCATTCTGGTGAGTGG 3' | 20bp |
| NbT16-F | 5' ATAGTACACCAAGACCCCGC 3' | 20bp | 1100bp | 957bp |
| NbT16-R | 5' TGGCATTTGTGTCATGGGGT 3' | 20bp |
| Nbh4-F | 5' AATGCTCGGCTGGCAACTC 3' | 19bp | 604bp | 228bp |
| Nbh4-R | 5' CCAGAGTATGTGAGCGAATGGA 3' | 22bp |

**S2 Table. Primers designed for analyzing presence/absence polymorphisms of MITEs based on *N. bombycis* CQ1 genomic sequences.**
