# Supplementary material for: Identification, Diversity and Evolution of MITEs in the Genomes of Microsporidian Nosema Parasites
Source: PLoS One. 2015 Apr 21;10(4):e0123170. doi: 10.1371/journal.pone.0123170 (PMC4405373; doi:10.1371/journal.pone.0123170)
Supplement: S3 Table — (DOC) [file pone.0123170.s012.doc]

**S3 Table. Characteristics of all MITE families in three genomes of *N. bombycis, N. antheraeae* and *N. ceranae.***

| **Family** | **Superfamily** | **Copy number** | **Full-length copy number** | **Length(bp)** | **Average AT content(%)** | **-ΔG(kcal/mol)** | **5 ’-flank < 300bp** | | **3 ’-flank < 300bp** | **CDS** | **Old name** | **Reference** |
| --- | --- | --- | --- | --- | --- | --- | --- | --- | --- | --- | --- | --- |
| ***Nosema bombcis*** | | |  |  |  |  |  |  | |  |  |  |
| *NbS1* | *Stowaway-like* | 9 | 8 | 237~287 | 75 | 45.70 | 1 | |  |  |  |  |
| *NbS2* | *Stowaway-like* | 4 | 3 | 140~146 | 69.72 | 27.80 |  | |  |  |  |  |
| *NbS3* | *Stowaway-like* | 6 | 6 | 217~242 | 60.83 | 47.20 | 1 | | 1 |  |  |  |
| *NbS4* | *Stowaway-like* | 10 | 7 | 265~277 | 59.56 | 53.00 |  | |  |  |  |  |
| *NbS5* | *Stowaway-like* | 17 | 5 | 165~177 | 70.11 | 38.90 |  | | 2 | 1 |  |  |
| *NbS6* | *Stowaway-like* | 3 | 3 | 228~248 | 70.78 | 90.50 |  | |  |  |  |  |
| *NbS7* | *Stowaway-like* | 15 | 7 | 680~721 | 73.85 | 185.40 |  | |  |  |  |  |
| *NbS8* | *Stowaway-like* | 3 | 3 | 327~328 | 71.65 | 78.00 |  | |  |  |  |  |
| *NbS9* | *Stowaway-like* | 8 | 6 | 579~684 | 68.9 | 188.60 | 1 | | 1 |  |  |  |
| *NbS10* | *Stowaway-like* | 11 | 11 | 261~313 | 63.55 | 70.10 |  | | 1 |  |  |  |
| *NbS11* | *Stowaway-like* | 7 | 3 | 260~285 | 70.36 | 82.50 |  | |  |  |  |  |
| *NbS12* | *Stowaway-like* | 19 | 4 | 274~348 | 61.24 | 74.26 | 1 | | 3 | 1 |  |  |
| *NbS13* | *Stowaway-like* | 9 | 8 | 759~764 | 62.35 | 202.10 |  | | 1 |  |  |  |
| *NbS14* | *Stowaway-like* | 31 | 5 | 281~349 | 58.7 | 90.60 | 1 | | 2 | 1 |  |  |
| *NbS15* | *Stowaway-like* | 10 | 3 | 1442~1456 | 78.11 | 346.30 |  | | 1 |  |  |  |
| *NbS16* | *Stowaway-like* | 31 | 22 | 266~339 | 70.09 | 68.40 | 1 | | 1 |  |  |  |
| *NbS17* | *Stowaway-like* | 8 | 7 | 572~635 | 69.1 | 124.60 | 1 | |  |  |  |  |
| *NbS18* | *Stowaway-like* | 12 | 3 | 800~886 | 65.54 | 163.20 | 1 | |  |  | NBME5 | Xu et al., 2010 |
| *NbS19* | *Stowaway-like* | 20 | 10 | 387~431 | 70.28 | 83.50 |  | |  |  |  |  |
| *NbS20* | *Stowaway-like* | 33 | 4 | 211~255 | 63.92 | 72.30 |  | | 2 |  |  |  |
| *NbS21* | *Stowaway-like* | 12 | 3 | 258~329 | 60.88 | 98.40 |  | |  |  |  |  |
| *NbS22* | *Stowaway-like* | 8 | 5 | 474~615 | 68.37 | 164.50 | 1 | |  |  |  |  |
| *NbS23* | *Stowaway-like* | 24 | 3 | 402~466 | 72.75 | 87.10 |  | | 1 |  |  |  |
| *NbS24* | *Stowaway-like* | 85 | 64 | 317~384 | 61.82 | 94.90 | 5 | | 8 | 3 | NBME3 | Xu et al., 2010 |
| *NbS25* | *Stowaway-like* | 18 | 4 | 419~477 | 72.65 | 85.90 | 2 | | 1 |  |  |  |
| *NbS26* | *Stowaway-like* | 6 | 4 | 405~446 | 72.56 | 80.10 |  | |  |  |  |  |
| *NbS27* | *Stowaway-like* | 9 | 3 | 747~889 | 70.14 | 174.10 |  | | 1 |  |  |  |
| *NbS28* | *Stowaway-like* | 5 | 5 | 665~773 | 73.58 | 133.30 |  | |  |  |  |  |
| *NbS29* | *Stowaway-like* | 4 | 4 | 712~728 | 69.29 | 152.20 | 1 | |  |  |  |  |
| *NbS30* | *Stowaway-like* | 29 | 17 | 312~379 | 69.79 | 108.40 |  | | 1 |  |  |  |
| *NbS31* | *Stowaway-like* | 8 | 4 | 655~796 | 67.8 | 187.90 | 1 | | 1 |  |  |  |
| *NbS32* | *Stowaway-like* | 8 | 6 | 454~571 | 68.73 | 149.50 |  | | 1 |  |  |  |
| *NbT1* | *Tourist-like* | 13 | 8 | 206~239 | 56.09 | 62.30 | 1 | |  |  |  |  |
| *NbT2* | *Tourist-like* | 8 | 7 | 176~207 | 59.13 | 36.30 |  | |  |  |  |  |
| *NbT3* | *Tourist-like* | 4 | 4 | 120~136 | 47.79 | 30.90 |  | | 2 |  |  |  |
| *NbT4* | *Tourist-like* | 11 | 5 | 146~153 | 60.81 | 21.00 |  | | 1 |  |  |  |
| *NbT5* | *Tourist-like* | 7 | 5 | 196~206 | 66.83 | 33.30 |  | | 2 |  |  |  |
| *NbT6* | *Tourist-like* | 23 | 4 | 558~593 | 70.66 | 103.10 |  | | 1 |  |  |  |
| *NbT7* | *Tourist-like* | 7 | 3 | 288~319 | 66.67 | 63.10 |  | | 1 |  |  |  |
| *NbT8* | *Tourist-like* | 8 | 5 | 169~181 | 59.66 | 50.90 |  | | 1 |  |  |  |
| *NbT9* | *Tourist-like* | 22 | 3 | 232~244 | 67.51 | 54.50 |  | | 1 |  |  |  |
| *NbT10* | *Tourist-like* | 14 | 7 | 210~223 | 55.66 | 69.20 | 2 | | 1 |  |  |  |
| *NbT11* | *Tourist-like* | 5 | 5 | 324~329 | 71.34 | 61.10 |  | |  |  |  |  |
| *NbT12* | *Tourist-like* | 10 | 5 | 695~710 | 66.19 | 170.50 | 3 | |  |  |  |  |
| *NbT13* | *Tourist-like* | 11 | 4 | 345~445 | 71.23 | 125.30 |  | |  |  |  |  |
| *NbT14* | *Tourist-like* | 21 | 8 | 220~319 | 63.64 | 95.90 |  | |  |  |  |  |
| *NbT15* | *Tourist-like* | 17 | 10 | 669~844 | 69.23 | 169.00 |  | |  | 1 |  |  |
| *NbT16* | *Tourist-like* | 25 | 18 | 924~961 | 66.56 | 197.60 | 1 | | 4 |  |  |  |
| *NbT17* | *Tourist-like* | 12 | 6 | 176~211 | 55.56 | 32.10 | 1 | | 2 |  |  |  |
| *NbT18* | *Tourist-like* | 4 | 4 | 612~616 | 71.9 | 134.40 |  | |  |  |  |  |
| *NbT19* | *Tourist-like* | 7 | 7 | 1051~1059 | 69.01 | 211.20 | 1 | |  | 2 |  |  |
| *NbN1* |  | 26 | 5 | 109~146 | 69.92 | 21.80 | 1 | | 2 |  |  |  |
| *NbN2* |  | 4 | 4 | 425~475 | 77.99 | 103.40 |  | | 1 |  |  |  |
| *NbN3* |  | 25 | 25 | 288~297 | 65.97 | 66.40 | 1 | | 1 |  |  |  |
| *NbN4* |  | 11 | 8 | 199~209 | 64.53 | 35.10 |  | | 1 |  |  |  |
| *NbN5* |  | 58 | 38 | 167~193 | 54.92 | 60.40 |  | | 2 |  | NBME2 | Xu et al., 2010 |
| *NbN6* |  | 16 | 3 | 192~221 | 47.39 | 77.30 | 2 | | 1 |  |  |  |
| *NbN7* |  | 15 | 7 | 381~399 | 68.83 | 85.30 | 1 | |  |  |  |  |
| *NbN8* |  | 31 | 5 | 181~240 | 54.39 | 47.36 |  | | 1 |  |  |  |
| *NbN9* |  | 3 | 3 | 1437~1466 | 79.06 | 332.26 |  | |  |  |  |  |
| *NbN10* |  | 8 | 3 | 416~477 | 75.05 | 73.80 |  | |  |  |  |  |
| *NbN11* |  | 10 | 5 | 411~483 | 72.65 | 93.20 |  | | 2 |  |  |  |
| *NbN12* |  | 21 | 7 | 583~746 | 68.54 | 147.60 | 1 | | 1 |  |  |  |
| *NbN13* |  | 15 | 9 | 233~245 | 58.4 | 82.50 |  | |  |  |  |  |
| *NbN14* |  | 7 | 4 | 137~147 | 49.65 | 24.30 |  | |  |  |  |  |
| *NbN15* |  | 25 | 9 | 122~155 | 59.6 | 32.90 | 4 | | 1 |  |  |  |
| *NbN16* |  | 13 | 13 | 273~275 | 70.61 | 61.60 |  | |  |  |  |  |
| *NbN17* |  | 9 | 7 | 222~233 | 64.32 | 47.70 | 1 | | 2 |  |  |  |
| *NbN18* |  | 5 | 4 | 547~646 | 63.23 | 170.10 |  | | 2 |  |  |  |
| *NbN19* |  | 26 | 9 | 192~208 | 65.13 | 41.80 | 1 | | 3 |  |  |  |
| *NbN20* |  | 9 | 3 | 226~245 | 53.51 | 64.50 |  | |  |  | NBME6 | Xu et al., 2010 |
| *NbN21* |  | 10 | 8 | 182~195 | 60.99 | 49.80 | 3 | | 1 |  |  |  |
| *NbN22* |  | 15 | 3 | 271~343 | 68.97 | 56.20 |  | |  |  |  |  |
| *NbN23* |  | 12 | 3 | 315~336 | 66.96 | 71.90 | 2 | |  |  |  |  |
| *NbN24* |  | 13 | 7 | 314~338 | 64.67 | 73.70 |  | |  |  |  |  |
| *NbN25* |  | 26 | 16 | 263~295 | 65.59 | 69.60 |  | | 3 |  |  |  |
| *Nbh1* | *hAT-like* | 54 | 41 | 244~280 | 60.93 | 58.30 | 1 | | 7 |  |  |  |
| *Nbh2* | *hAT-like* | 76 | 6 | 176~215 | 59.35 | 57.40 | 12 | | 2 | 2 |  |  |
| *Nbh3* | *hAT-like* | 3 | 3 | 626~633 | 66.88 | 136.20 |  | |  |  |  |  |
| *Nbh4* | *hAT-like* | 122 | 112 | 199~248 | 53.28 | 76.70 | 18 | | 5 | 10 | NBME1 | Xu et al., 2010 |
| *Nbh5* | *hAT-like* | 20 | 3 | 259~318 | 62.03 | 93.20 |  | | 2 |  |  |  |
| *Nbh6* | *hAT-like* | 21 | 17 | 272~336 | 61.98 | 89.50 |  | | 1 |  |  |  |
| *Nbh7* | *hAT-like* | 9 | 6 | 834~1040 | 70.22 | 185.20 |  | | 1 |  |  |  |
| *Nbh8* | *hAT-like* | 4 | 4 | 680~710 | 75.5 | 116.10 | 1 | |  |  |  |  |
| *NbMe1* | *Merlin-like* | 12 | 6 | 350~391 | 67.3 | 94.40 |  | |  |  |  |  |
| *NbMe2* | *Merlin-like* | 15 | 9 | 272~322 | 66.34 | 84.10 |  | |  |  |  |  |
| *NbMe3* | *Merlin-like* | 12 | 5 | 458~586 | 63.27 | 126.30 |  | |  |  |  |  |
| *NbMe4* | *Merlin-like* | 9 | 5 | 318~388 | 72.42 | 83.60 |  | |  |  |  |  |
| *NbMu1* | *Mutator-like* | 9 | 4 | 75~136 | 52.27 | 38.00 |  | | 1 |  |  |  |
| ***Nosema antheraeae*** | | |  |  |  |  |  | |  |  |  |  |
| *NaS1* | *Stowaway-like* | 3 | 3 | 153~171 | 72.21 | 23.36 |  | | 2 |  |  |  |
| *NaS2* | *Stowaway-like* | 8 | 7 | 120~152 | 70.97 | 27.60 | 4 | | 1 |  |  |  |
| *NaS3* | *Stowaway-like* | 10 | 3 | 68~85 | 74.28 | 13.60 | 4 | | 2 |  |  |  |
| *NaT1* | *Tourist-like* | 15 | 12 | 160~176 | 69.45 | 32.30 | 2 | | 4 |  |  |  |
| *NaT2* | *Tourist-like* | 5 | 4 | 119~151 | 68.18 | 29.30 | 1 | | 1 |  |  |  |
| *NaT3* | *Tourist-like* | 9 | 4 | 200~214 | 69.46 | 32.10 |  | | 1 | 1 |  |  |
| *NaT4* | *Tourist-like* | 8 | 6 | 137~151 | 67.43 | 22.70 | 1 | |  |  |  |  |
| *NaT5* | *Tourist-like* | 7 | 6 | 68~75 | 77.49 | 7.50 | 1 | | 5 | 1 |  |  |
| *NaT6* | *Tourist-like* | 9 | 4 | 148~182 | 63.34 | 37.90 |  | | 6 |  |  |  |
| *NaT7* | *Tourist-like* | 5 | 3 | 173~186 | 70.7 | 28.90 |  | | 1 |  |  |  |
| *NaT8* | *Tourist-like* | 7 | 7 | 176 | 62.5 | 45.10 |  | | 3 |  |  |  |
| *NaN1* |  | 5 | 4 | 183~193 | 57.41 | 62.30 |  | |  |  |  |  |
| *NaN2* |  | 13 | 6 | 65~76 | 74.73 | 4.90 | 3 | | 4 | 2 |  |  |
| *NaN3* |  | 13 | 9 | 140~161 | 70.89 | 31.30 | 2 | | 1 | 1 |  |  |
| *NaN4* |  | 12 | 4 | 203~220 | 56.44 | 55.70 | 1 | |  | 2 |  |  |
| *NaN5* |  | 12 | 8 | 196~210 | 61.98 | 55.30 | 1 | |  |  |  |  |
| *Nah1* | *hAT-like* | 8 | 3 | 231~255 | 67.62 | 55.80 | 1 | | 2 |  |  |  |
| ***Nosema ceranae*** | | |  |  |  |  |  | |  |  |  |  |
| *NcS1* | *Stowaway-like* | 12 | 3 | 110~149 | 72.3 | 44.10 |  | | 1 | 1 |  |  |
| *NcT1* | *Tourist-like* | 5 | 3 | 97~112 | 75.37 | 12.80 | 2 | |  |  |  |  |
| *NcT2* | *Tourist-like* | 7 | 3 | 293~313 | 70.58 | 61.10 |  | | 1 |  |  |  |
| *NcT3* | *Tourist-like* | 34 | 6 | 97~120 | 69.97 | 20.50 | 4 | | 10 | 1 |  |  |
| *NcN1* |  | 25 | 17 | 273~292 | 64.77 | 55.20 |  | | 3 | 1 |  |  |
