# Supplementary material for: Identification, Diversity and Evolution of MITEs in the Genomes of Microsporidian Nosema Parasites
Source: PLoS One. 2015 Apr 21;10(4):e0123170. doi: 10.1371/journal.pone.0123170 (PMC4405373; doi:10.1371/journal.pone.0123170)
Supplement: S5 Table — (DOC) [file pone.0123170.s014.doc]

**S5 Table. Chi-square test of biased insertion of MITE besides gene regions in *N. bombycis*, *N. antheraeae* and *N. ceranae* genomes.**

**5’-flanking regions<300bp; 3’-flanking regions<300bp**

|  | Experimental | | Control | | *chi-square* test |
| --- | --- | --- | --- | --- | --- |
|  | Gene region | Intergenic | Gene region | intergenic | *P* values |
| *Nb* | 189(12.7) | 1301 | 276(13.8) | 1724 | 0.337 |
| *Na* | 61(40.9) | 88 | 672(33.6) | 1328 | 0.068 |
| *Nc* | 24(28.9) | 59 | 426(21.3) | 1574 | 0.195 |

**5’-flanking regions<200bp; 3’-flanking regions<200bp**

|  | Experimental | | Control | | *chi-square* test |
| --- | --- | --- | --- | --- | --- |
|  | Gene region | Intergenic | Gene region | intergenic | *P* values |
| *Nb* | 165(11.1) | 1325 | 213(10.7) | 1787 | 0.69 |
| *Na* | 47(31.5) | 102 | 489(24.4) | 1511 | 0.054 |
| *Nc* | 17(20.5) | 66 | 267(13.4) | 1733 | 0.064 |

**5’-flanking regions<100bp; 3’-flanking regions<100bp**

|  | Experimental | | Control | | *chi-square* test |
| --- | --- | --- | --- | --- | --- |
|  | Gene region | Intergenic | Gene region | intergenic | *P* values |
| *Nb* | 92(6.2) | 1398 | 114(5.7) | 1886 | 0.556 |
| *Na* | 27(18.1) | 122 | 268(13.4) | 1725 | 0.106 |
| *Nc* | 8(9.6) | 75 | 107(5.4) | 1893 | 0.094 |

5’-flanking regions=0bp; 3’-flanking regions=0bp

|  | Experimental | | Control | | *chi-square* test |
| --- | --- | --- | --- | --- | --- |
|  | Gene region | Intergenic | Gene region | intergenic | *P* values |
| *Nb* | 21(1.4) | 1469 | 24(1.2) | 1976 | 0.588 |
| *Na* | 7(4.7) | 142 | 53(2.6) | 1947 | 0.143 |
| *Nc* | 3(3.6) | 80 | 41(1.8) | 1964 | 0.223 |

Nb: *Nosema bombycis*, Na: *Nosema antheraeae*, Nc: *Nosema ceranae*. Number in each bracket represent the percentage of the specific insertion sites occupy the total insertion sites. Gene regions contain CDSs, 5’-flanking regions and 3’-flanking regions.
