# Supplementary material for: Identification, Diversity and Evolution of MITEs in the Genomes of Microsporidian Nosema Parasites
Source: PLoS One. 2015 Apr 21;10(4):e0123170. doi: 10.1371/journal.pone.0123170 (PMC4405373; doi:10.1371/journal.pone.0123170)
Supplement: S6 Table — (DOC) [file pone.0123170.s015.doc]

**S6 Table. The protein coding regions associated with MITE insertion in *N. bombycis.***

| CDS name | CDS Length (bp) | MITE element name | Insertion site in CDS |
| --- | --- | --- | --- |
| *NBO_298g0003* | 1891 | *Nbh4* | *791~1030* |
| *NBO_27g0065* | 4759 | *Nbh4* | *3305~3544* |
| *NBO_6gi004* | 1702 | *Nbh4* | *1382~1608* |
| *NBO_29g0016* | 976 | *Nbh4* | *361~586* |
| *NBO_1078gi001* | 1890 | *Nbh4* | *712~938* |
| *NBO_41g0008* | 737 | *Nbh4* | *359~585* |
| *NBO_55g0015* | 2336 | *Nbh4* | *1839~2063* |
| *NBO_411gi001* | 1059 | *Nbh4* | *976~1200* |
| *NBO_460gi001* | 1347 | *Nbh4* | *1256~1496* |
| *NBO_1345gi001* | 765 | *Nbh4* | *667~893* |
| *NBO_1060gi001* | 2190 | *Nbh2* | *2106~2317* |
| *NBO_10g0007* | 1449 | *Nbh2* | *827~1039* |
| *NBO_61g0010* | 5894 | *NbT15* | *1440~2279* |
| *NBO_83g0001* | 4385 | *NbT19* | *1562~2613* |
| *NBO_376gi001* | 915 | *NbT19* | *833~1888* |
| *NBO_10gi001* | 501 | *NbS5* | *410~576* |
| *NBO_389g0002* | 2342 | *NbS12* | *1750~2092* |
| *NBO_380gi001* | 801 | *NbS14* | *757~1091* |
| *NBO_2g0009* | 2916 | *NbS24* | *1169~1551* |
| *NBO_602gi001* | 1323 | *NbS24* | *1211~1591* |
| *NBO_283gi001* | 1899 | *NbS24* | *643~1023* |
